# Metabolic Adaptations of White Lupin Roots and Shoots under Phosphorus Deficiency

**DOI:** 10.3389/fpls.2015.01014

**Published:** 2015-11-24

**Authors:** Julia Müller, Victoria Gödde, Karsten Niehaus, Christian Zörb

**Affiliations:** ^1^Crop Product Quality, Institute of Crop Science, University of HohenheimStuttgart, Germany; ^2^Centre for Biotechnology–CeBiTec, Faculty of Biology, Bielefeld UniversityBielefeld, Germany

**Keywords:** white lupin, cluster roots, metabolic profiling, phosphorus, sugar signaling

## Abstract

White lupin (*Lupinus albus* L.) is highly adapted to phosphorus-diminished soils. P-deficient white lupin plants modify their root architecture and physiology to acquire sparingly available soil phosphorus. We employed gas chromatography–mass spectrometry (GC-MS) for metabolic profiling of P-deficient white lupins, to investigate biochemical pathways involved in the P-acquiring strategy. After 14 days of P-deficiency, plants showed reduced levels of fructose, glucose, and sucrose in shoots. Phosphorylated metabolites such as glucose-6-phosphate, fructose-6-phosphate, myo-inositol-phosphate and glycerol-3-phosphate were reduced in both shoots and roots. After 22 days of P-deficiency, no effect on shoot or root sugar metabolite levels was found, but the levels of phosphorylated metabolites were further reduced. Organic acids, amino acids and several shikimate pathway products showed enhanced levels in 22-day-old P-deficient roots and shoots. These results indicate that P-deficient white lupins adapt their carbohydrate partitioning between shoot and root in order to supply their growing root system as an early response to P-deficiency. Organic acids are released into the rhizosphere to mobilize phosphorus from soil particles. A longer period of P-deficiency leads to scavenging of P_i_ from P-containing metabolites and reduced protein anabolism, but enhanced formation of secondary metabolites. The latter can serve as stress protection molecules or actively acquire phosphorus from the soil.

## Introduction

Phosphorus (P), in the form of inorganic phosphate (P_i_), is an essential plant macronutrient and one of the most limiting factors in plant growth. Phosphorus is a structural element in nucleic acids and in the phospholipids that are components of biomembranes. Phosphoesters are essential for cellular energy transfer, and by means of protein phosphorylation and dephosphorylation P_i_ is also a key regulator of signal transduction. Although the P-concentration in most soils is adequate for plant nutrition, P_i_ availability is often limited. The rapid formation of organic complexes and the inorganic fixation of free P_i_ in the soil are responsible for the low P-availability. Plants use morphological, physiological, molecular and biochemical adaptations to thrive in such environments. White lupins form densely clustered lateral rootlets, the so-called proteoid or cluster roots (CRs), when exposed to P-deficiency. These roots are a common characteristic of many members of the Proteaceae family, which live on nutrient-poor soils of the southern hemisphere, but have also evolved in various species of other plant families (Dinkelaker et al., 1995; Lamont, 2003; Shane and Lambers, 2005). White lupin is the only CR-forming crop and has therefore become a model plant for studying morphological and biochemical adaptations to P-deficiency. On the one hand, the formation of CRs increases the surface of the root system, giving access to a larger soil volume. On the other hand, the finding that CRs can acquire 10 times more P than non-CRs (Jeschke and Pate, 1995) is not only attributable to their morphology. Lupin CRs exude striking amounts of organic acids (mainly citrate and malate) and protons into the rhizosphere (Dinkelaker et al., 1989; Neumann and Römheld, 1999). These substances lower the pH of the soil solution in the rhizosphere and liberate P_i_ from soil surfaces by ligand exchange or from complexes with aluminum, iron, or calcium ions. Moreover, CRs secrete an acid phosphatase and so access the soil organic P pool by hydrolyzing P-monoesters (Tadano and Sakai, 1991; Gilbert et al., 1999; Wasaki et al., 2003). A variety of physiological mechanisms is linked to the increased ability of CRs with regard to exudation and P-uptake. These include an enhancement of the citrate anion and proton transport across the plasma membrane (Neumann et al., 1999; Penaloza et al., 2002; Yan et al., 2002), the induction of a high affinity P transport system (Liu et al., 2001) and metabolic alterations.

When exposed to P-deprivation, plants alter their metabolism to scavenge and conserve internal P_i_. For example, phospholipids in biomembranes can be replaced by sulfo- and galactolipids, and plants can use metabolic bypass reactions that depend on inorganic pyrophosphate (PP_i_) instead of P_i_ (Plaxton and Tran, 2011). One of those bypasses is a PP_i_-dependent glycolysis. This strategy enables the plants to maintain the carbon flow down to the citric acid cycle. Moreover, a non-energy-conserving pathway of the mitochondrial electron transport chain involves an alternative oxidase (AOX). Therefore, respiration can be maintained, even when ADP and P_i_ levels are low. The replacement of phospholipids, the induction of a PP_i_-dependent glycolysis, and the respiratory AOX pathway are all established alterations found in white lupin CRs (Uhde-Stone et al., 2003; Florez-Sarasa et al., 2014; Wang et al., 2014). Moreover, CRs have a strongly increased citrate synthesis, whereas the turnover of citrate to isocitrate is inhibited. Increased synthesis and inhibited turnover together lead to an accumulation of citrate, that will then be released into the rhizosphere (Neumann et al., 1999; Neumann and Martinoia, 2002; Kihara et al., 2003).

The enhancement of phosphate acquisition and of the efficiency of the phosphate utilization of crops are pressing research subjects. Rock phosphates, the source of low-cost P-fertilizers, are predicted to be depleted by the end of the 21st century (Vance, 2001). During the same time, the world’s population could increase to over 12 billion (Gerland et al., 2014). This makes it necessary to use additional land for agriculture, even if it is of only low or marginal fertility. White lupin is a crop plant with a highly effective phosphorus acquisition strategy. A complete elucidation of this strategy could contribute to defining targets for breeding other P-efficient crops and help in further understanding the physiology of plant P acquisition.

Metabolic profiling is considered a major tool in studying plant stress responses (Guy et al., 2008; Shulaev et al., 2008; Král’ová et al., 2012). A variety of abiotic stresses, including shortage in nutrients, have been investigated by using metabolomics (Obata and Fernie, 2012; Jorge et al., 2015). Metabolic profiling studies under P-deficiency are available for a number of plant species, including common bean (Huang et al., 2008), *Arabidopsis thaliana* (Pant et al., 2014), and maize (Ganie et al., 2015). The latter studies found an increase in di- and trisaccharides, an accumulation of amino acids, and a decrease in P-containing metabolites in the P-deprived plants. However, these observations do not represent a general plant response to P-deficiency. The perennial plant *Eucalyptus globulus* seems to possess a different adaptation strategy to P-deficiency, which does not involve strong changes in the levels of sugars, amino acids, or organic acids (Warren, 2011). The effect of short-term P-deficiency on the metabolism of perennial ryegrass was investigated by Byrne et al. (2011). They showed that metabolic alterations, such as the replacement of phospholipids and the induction of glycolytic bypasses, are initiated after only 24 h of P-deprivation. Lipidomics identified a new class of lipids with protective functions against phosphorus depletion (Okazaki et al., 2013).

Previous investigations concerning metabolites in white lupins have mainly focused on those metabolites involved in root exudation and on CRs (Johnson et al., 1994; Massonneau et al., 2001; Kihara et al., 2003; Uhde-Stone et al., 2003). Until now, a metabolite profile of P-deficient white lupins, covering a wide range of metabolites and involving shoots and roots, was missing. Our non-targeted metabolic profiling aims to fill this gap and integrates shoot and root responses of P-deficient white lupins. Plants in two different developmental stages were investigated to gain information about the early response to P-deficiency as well as about the metabolic adaptations to longer periods of P-deficiency. Thus, our investigation renders additional insights into metabolic changes of P-deficient white lupin plants.

## Materials and Methods

### Plant Cultivation

Seeds of white lupin (*Lupinus albus* L. cv. Feodora, Südwestsaat GbR, Rastatt, Germany) were soaked in aerated 1 mM CaSO_4_ for 1 day and germinated at room temperature in the dark between two layers of filter paper moistened with 1 mM CaSO_4_. After 4 days, seedlings were transferred to pots containing 4.5 L aerated nutrient solution. Per treatment, four biological replicates with four plants per pot were cultivated. The concentration of the nutrient solution was one-fourth at the beginning and was increased to one-half after 2 days and to full strength after another 2 days. The full-strength nutrient solution had the following composition: 0.5 mM Ca(NO_3_)_2_, 1.75 mM K_2_SO_4_, 0.25 mM KCl, 1.25 mM MgSO_4_, 25 μM H_3_BO_3_, 1.5 μM MnSO_4_, 1.5 μM ZnSO_4_, 0.5 μM CuSO_4_, 0.025 μM (NH_4_)_6_Mo_7_O_24_, 20 μM Fe(III)-EDTA (according to Yan et al., 2002). The control plants received additional 0.5 mM KH_2_PO_4_. The pH in the pots was daily adjusted to 6.0, and the nutrient solution was changed every 3 days. Plants were grown in a growth room under controlled conditions of a day/night cycle of 16 h/8 h at 24°C/21°C and a light intensity of approximately 280 μmol photons m^2^ s^-1^ at shoot height.

### Plant Harvest

To investigate the early response of white lupin to P-deficiency, two plants per pot were removed after 14 days in hydroponics, washed briefly in distilled water, separated into shoots, non-CRs, and CRs, and frozen in liquid nitrogen. The remaining two plants per pot were grown for a total of 22 days in hydroponics and then harvested in the same way. For metabolite extraction, the plant material was ground to a powder in liquid nitrogen and freeze-dried. For phosphorus analysis, plant material was dried at 70°C and ground to powder. The total phosphorus concentration in the dry matter was determined by using the vanado-molybdate method (Gericke and Kurmies, 1952).

### Metabolite Extraction

Metabolites were extracted from 10 mg material in a Precellys24 Instrument (Peqlab, Erlangen, Germany) with 1 mL 80% methanol, containing 10 mM ribitol as the internal standard, and 1 mm zirconia beads (Roth, Karlsruhe, Germany). Samples were treated three times at 6.5 m/s for 45 s. After 20 min of centrifugation at 15,000 × *g* at room temperature, 375 μl of the clear supernatant was transferred to 1 mL glass vials (Supelco, Bellefonte, California) and evaporated in a nitrogen stream. Metabolites were derivatized with 100 μL methoxylamine hydrochloride in pyridine (20 mg/mL; g/v) for 90 min at 37°C and with 100 μL MSTFA for 30 min at 37°C. All chemicals and standard compounds were purchased from Sigma–Aldrich-Fluka (Taufkirchen, Germany), Merck (Darmstadt, Germany), or Macherey-Nagel (Düren, Germany).

### GC-MS Analysis

Sample volumes of 1 μL were analyzed with a Trace GC gas chromatograph coupled to a PolarisQ ion trap mass spectrometer equipped with an AS2000 auto sampler (Thermo Electron, Dreieich, Germany). Derivatized metabolites were evaporated at 250°C in the splitless mode and separated on a 30 m × 0.25 mm RTX-5MS capillary column with a 0.25 mm coating equipped with an integrated 10 m guard column (Restek, Bad Homburg, Germany). Helium carrier gas flow was adjusted to 1 mL/min. The interface temperature was set to 250°C and the ion source temperature to 220°C. Oven temperature was kept constant for 3 min at 80°C and subsequently raised to 325°C at 5°C/min. The system was equilibrated for 2 min at 80°C after each analysis. Mass spectra were recorded at 1 scan/s with a scanning range of 50 to 750 m/z. Metabolites were identified by comparison with pure standards (Sigma–Aldrich) and by using the NIST 2005 database (NIST, Gaithersburg, MD, USA). In addition, the freely available Golm Metabolome Database (Kopka et al., 2005) was of particular help in identifying several metabolites. All identified compounds matched the references by mass spectral data and chromatographic retention time. Relative levels of selected metabolites were determined automatically by integrating the peak areas of selective ions (Fiehn et al., 2000) with the processing setup implemented in Xcalibur 1.4 software (Thermo Electron, Dreieich, Germany). Relative response ratios were calculated by normalizing the respective peak areas to the peak area of the internal standard ribitol and dividing the value by the dry weight of the sample. Measurements were performed in technical duplicates for each of the four biological replicates of control plants and P-deprived plants.

### Data Analyses and Visualization

Principal component analysis (PCA) was carried out by using the program MeltDB (Neuweger et al., 2008). For heatmaps and statistics, data were log_10_-transformed and centered (van den Berg et al., 2006). For root metabolites, one-way-ANOVA and Tukey’s test (*P* ≤ 0.05) were performed with SigmaPlot 11 (Systat Software, San Jose, USA). For shoot metabolites and P-concentrations, Student’s *t*-test (*P* ≤ 0.05) was performed with Microsoft Excel (2010, Microsoft Corporation, Redmont, WA, USA). The relative mean responses of metabolites and the results of statistics are available in supplementary Tables 1 and 2. Heatmaps were created with the MultiExperiment Viewer (MeV 4.9, http://www.tm4.org/mev.html) by using Pearson’s correlation and complete linkage. For the metabolic map, untransformed mean values were employed to calculate -P/+P ratios.

## Results

### Growth and P Nutrition Responses to P Starvation

**FIGURE 1:**
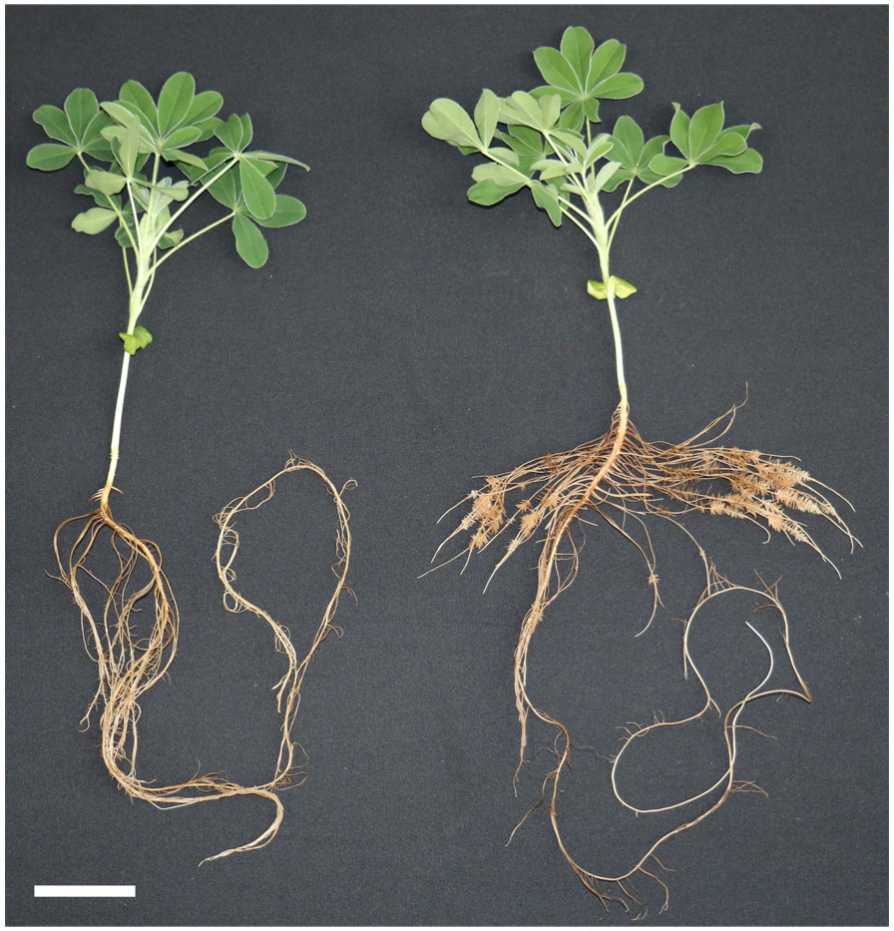
**Effect of phosphate concentration on plant growth of white lupin**. Plants were grown in hydroponics for 22 days with 0.5 mM KH_2_PO_4_ **(left)** or 0 mM KH_2_PO_4_ **(right)**. P-deficient plants developed a larger root system with cluster roots (CRs). Scale bar = 5 cm.

Lupin plants were grown from seeds either without a phosphorus supply (-P) or with 0.5 mM KH_2_PO_4_ (+P, control) in the nutrient solution (**Figure 1**). During cultivation, P-deficient plants developed a significantly higher root mass and CRs, whereas shoot growth was not significantly affected (**Table 1**). Phosphorus concentrations in the dry matter of shoots and roots clearly revealed the P-deficient status of those plants grown with no phosphorus supply. The P-deficient plants had a shoot P concentration of only one fourth of the control plants (**Table 1**). The root P concentration of P-sufficient plants exhibited high values, which might have resulted from apoplastic phosphorus residues that could not be removed by washing the roots in distilled water. However, shoots of P-supplied plants showed phosphorus concentrations in an optimal range (**Table 1**). For optimal growth, a P-concentration between 3 and 5 mg/g dry weight is required (Marschner and Marschner, 2012). Shoot P-concentrations below 2–3 mg/g dry weight generally induce the formation of CRs in white lupins (Li et al., 2008). P-deficient lupin plants in our investigation had much lower shoot P concentrations, but this did not affect their shoot biomass negatively. Therefore, any discovered metabolite change was not a result of diminished growth, but a true physiological response to a shortage in phosphorus.

**Table 1:** Fresh weight and phosphorus (P) concentrations of white lupin plants grown with and without phosphorus supply.

	Fresh weight	P-concentration
	(g) ± SD	(mg/g DM) ± SD
Shoot control	18.78 ± 1.57	4.95 ± 0.15
Shoot -P	16.38 ± 2.12	1.13 ± 0.15
Root control	14.70 ± 0.55	8.37 ± 0.27
Root -P	18.14 ± 1.33^∗^	1.69 ± 0.19

*Data are from plants grown hydroponically for 22 days. Four plants were pooled as one sample, *n* = 4. DM, dry matter; SD, standard deviation; ^∗^significantly different from control*.

### Metabolic Alterations during Phosphorus Deprivation

A GC-MS-based metabolic profiling method was used to investigate metabolites in P-deficient and P-sufficient shoots, non-CRs, and CRs at two different time points. An early time point (14 days of cultivation) was chosen for the first harvest. At that time, the P-deficient plants had just begun to show P-deficiency symptoms by forming their first CRs. Another set of samples was taken after 22 days of cultivation, when the P-deficient plants had developed a root system with numerous CRs (**Figure 1**).

**FIGURE 2:**
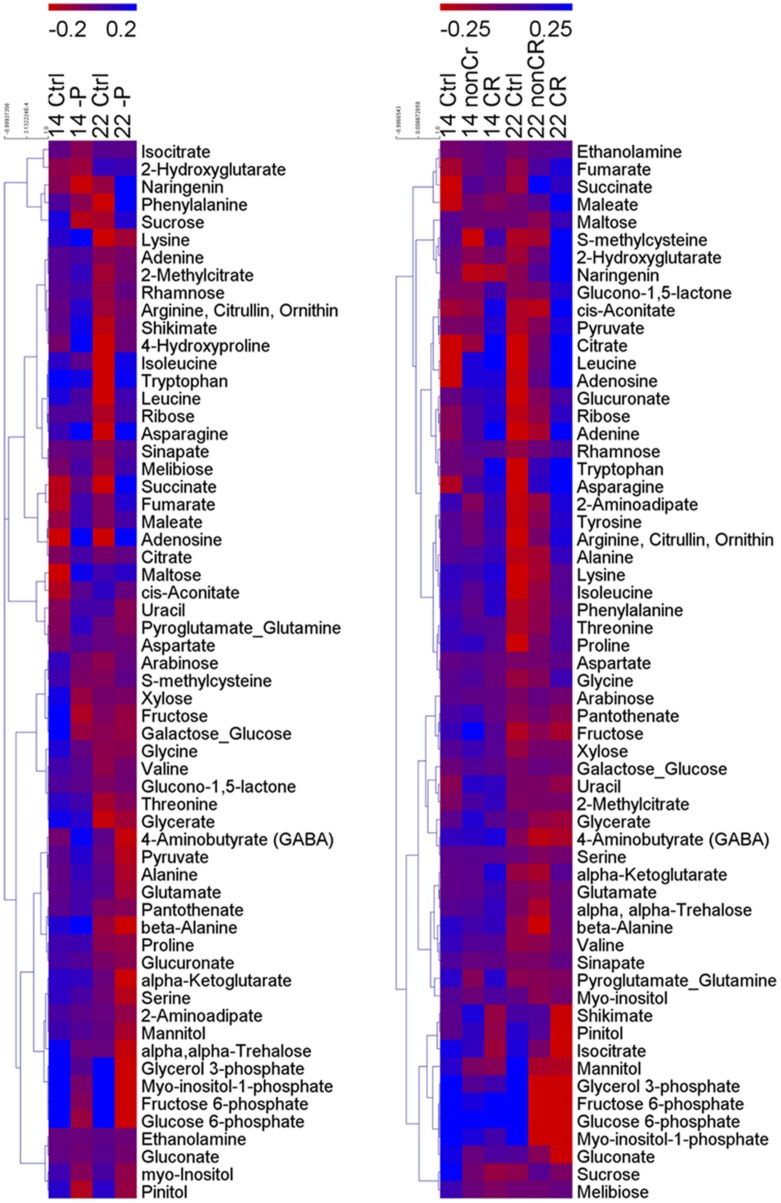
**Clustered heatmaps of metabolites of shoots **(left)** and roots **(right)****. Individual metabolites are represented by rows, and nutritional status and plant age are represented by columns. 14, 14-day old plants; 22, 22 days old plants; Ctrl, control plants; -P, P-deficient shoots; non-CR, non-cluster roots of P-deficient plants; CR, cluster roots of P-deficient plants. Heat map visualization of differences in metabolites is based on log10-transformed metabolite concentrations. Bluish colors indicate increased concentration levels of metabolites, reddish colors decreased metabolite levels. *n* = 4.

**FIGURE 3:**
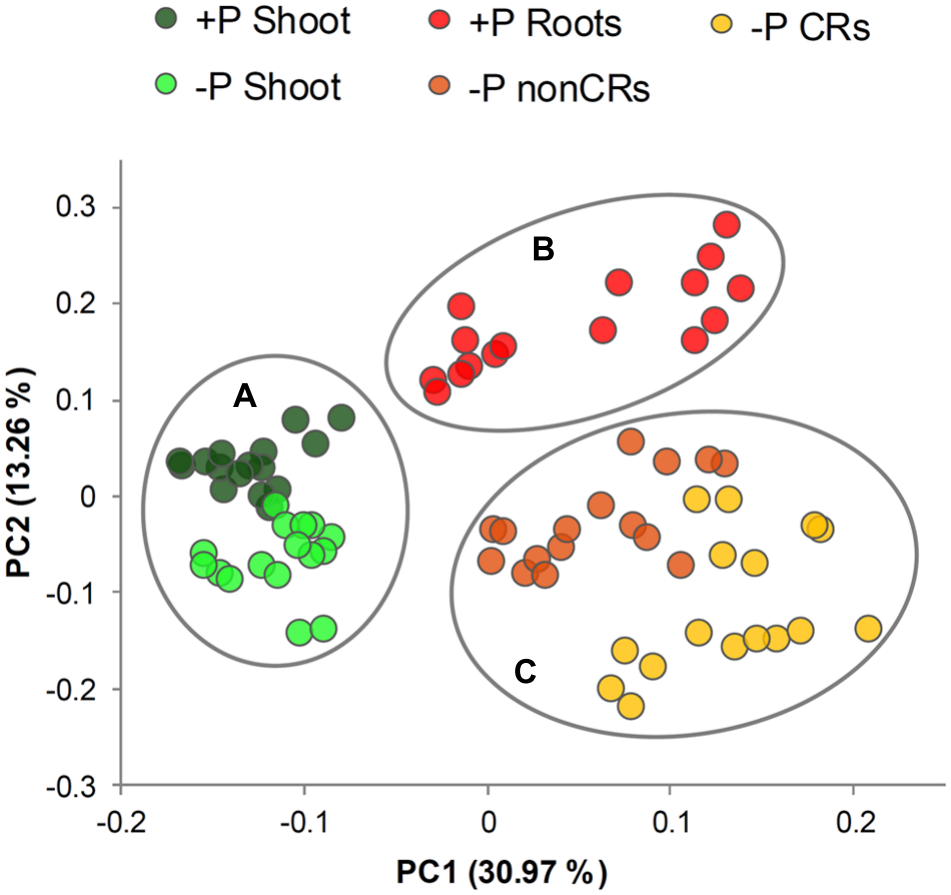
**Plot of principal component (PC) scores generated from relative metabolite concentrations in 14- and 22-day-old white lupin plants (*n* = 4)**. Plant organs showing cluster patterns are marked with circles. **(Circle A)** shoots of P-sufficient and P-deficient plants, **(circle B)** roots of P-sufficient plants, **(circle C)** non-cluster roots and cluster roots of P-deficient plants.

A total of 75 metabolites were measured in the shoots and roots of the lupins. Among them, 60 metabolites were detectable in the shoots and roots. Heatmaps provided an overview of the normalized mean values of these metabolites (**Figure 2**). A PCA was performed on the dataset. PCA is a tool to simplify complex data and to identify patterns in a dataset. Clusters of related samples are revealed when plotting each sample in a space that is formed by those variables (main components) that result in the largest sample separation of the whole dataset. As visualized in **Figure 3**, three different clusters were distinguished by the PCA. Shoot (circle A) and root (circles B and C) samples were clearly separated by principal component one. Principal component two separated P-sufficient and P-deficient plant material. This second separation was strict for root metabolites, as there were clearly two independent clusters for P-sufficient (circle B) and P-deficient roots (circle C). Moreover, P-deficient and P-sufficient shoots were separated by PCA, but overlapping areas occurred between these clusters (circle A).

**FIGURE 4:**
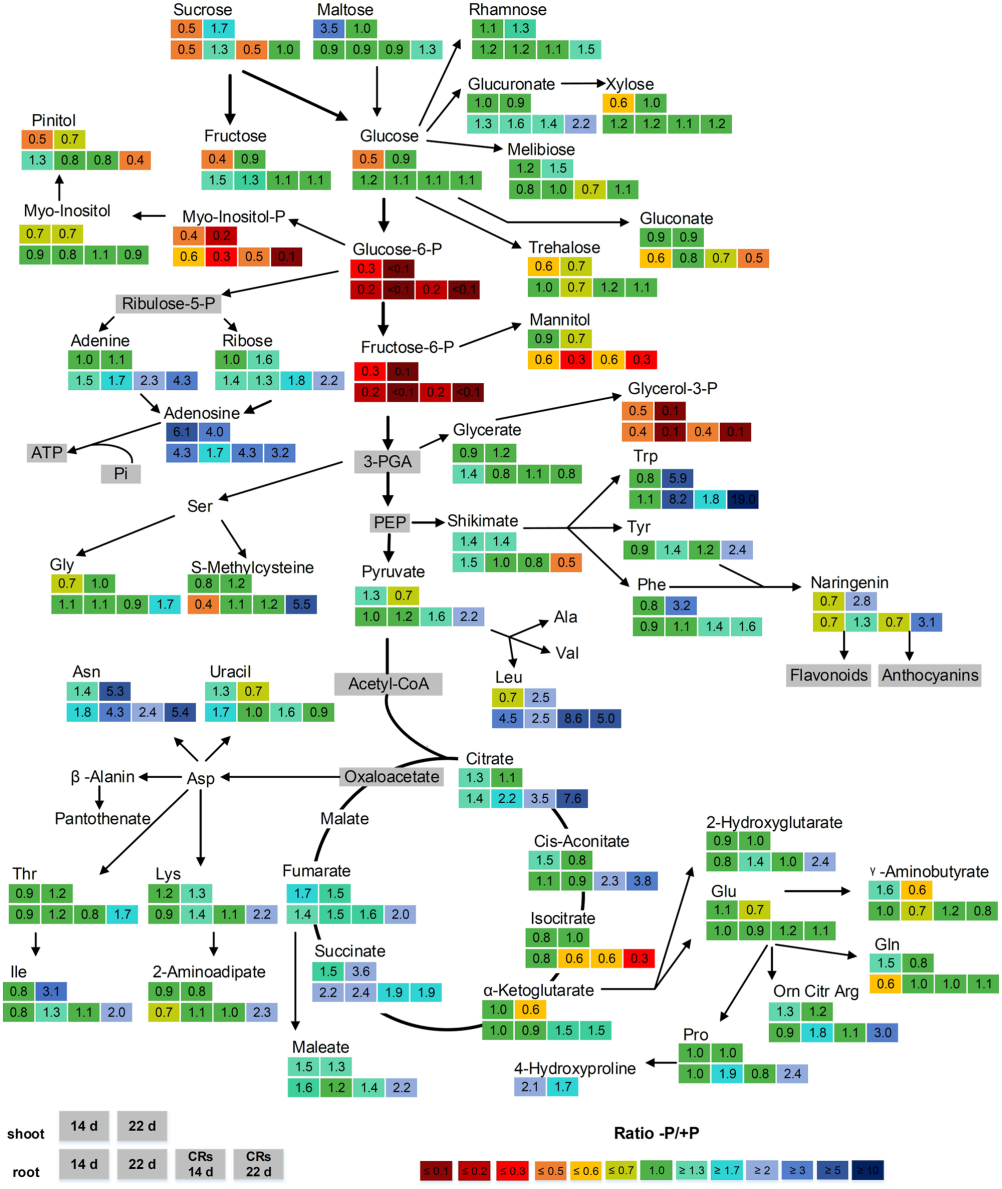
**Effect of P supply on the levels of metabolites from shoots and roots of 14- and 22-day-old white lupin plants**. Relative ratios (-P/+P), *n* = 4, are shown for metabolites with significant increases or decreases of at least 30% (1.3-fold/0.7-fold). Metabolites in gray boxes were not determined. The upper two boxes show the -P/+P ratio of shoots after 14 days (left box) and 22 days (right box). The lower four boxes show the -P/+P ratio of roots. From left to right: non-cluster roots after 14 days, non-cluster roots after 22 days, cluster roots after 14 days, cluster roots after 22 days.

The relative concentration of metabolites during P deficiency (-P) compared with P-sufficient (+P) plants was calculated as a response ratio (-P/+P). The -P/+P ratios for metabolite levels with statistically significant increases or decreases of at least 30% are summarized in **Figure 4**. The following paragraphs refer to **Figure 4** and describe selected metabolites in detail. Shoot-derived carbohydrates are a carbon source required for growth and development in every plant. These carbohydrates can also act as signaling molecules that promote or inhibit growth (Hammond and White, 2008). These functions make carbohydrates interesting for the investigation of abiotic stress responses in lupin. The sugars sucrose (0.5-fold), fructose (0.4-fold), and glucose (0.5-fold) were significantly reduced in shoots after 14 days of P-deficiency. Further, in 14-day-old non-CRs and CRs, the levels of sucrose decreased by half (0.5-fold). In contrast, fructose and glucose levels in non-CRs and CRs were not affected after 14 days of growth. In 22-day-old plants, neither shoot nor roots exhibited a change in their levels of sucrose, glucose, or fructose. The concentration of maltose, a degradation product of starch, was increased 3.5-fold in 14-day-old shoots but remained unchanged compared with control plants in 22-day-old shoots. In non-CRs and CRs, maltose levels remained unchanged at both time points.

The phosphorylated metabolites glucose-6-phosphate (Glc6P), fructose-6-phosphate (Fru6P), glycerol-3-phosphate (G3P), and, to a lesser extent, myo-inositol-phosphate (myo-inositol-P), were strongly reduced in P-deficient shoots and roots. This reduction was intensified with the duration of phosphorus deficiency. Whereas 14-day-old shoots and roots exhibited reductions of between 0.6-fold (myo-inositol-P) and 0.2-fold (Glc6P, Fru6P), the 22-day-old plants had levels reduced to less than 0.1-fold in Glc6P and Fru6P. Glc6P and Fru6P are intermediates during glycolysis and therefore are essential steps in carbohydrate metabolism. Moreover, G3P has a crucial role in plant cells, being a component of the glycerophospholipids of biomembranes.

Several organic acids of the tricarboxylic acid (TCA) cycle exhibited elevated levels. The strongest increase was found in concentrations of citrate in CRs (increase of 3.5-fold after 14 days and 7.6-fold after 22 days), followed by *cis*-aconitate (increase of 2.3-fold after 14 days and 3.8-fold after 22 days). This result was not surprising, as white lupin CRs accumulate large amounts of citrate and exude them into the rhizosphere to mobilize P. The tricarboxylic acid cycle of CRs is responsible for the supply of CRs with citrate. Whereas citrate and *cis*-aconitate accumulated in CRs, levels of isocitrate were reduced (0.6-fold after 14 days and 0.3-fold after 22 days). Succinate exhibited elevated levels in all P-deficient tissues compared with control plants, whereas fumarate was significantly increased in both shoots and CRs.

About half of the measured amino acids were clearly increased in P-deficient tissue. Strong reactions in both shoots and roots were found in 22-day-old plants, namely in tryptophan, followed by asparagine and leucine. Tryptophan was increased 5.9-fold in shoots, 8.2-fold in non-CRs, and 19-fold in CRs. Asparagine was increased 5.3-fold in shoots, 4.3-fold in non-CRs, and 5.4-fold in CRs. Leucine was increased 2.5-fold in shoots, 2.5-fold in non-CRs, and 5-fold in CRs. Shikimate, a precursor of the aromatic amino acids phenylalanine, tryptophan, and tyrosine, was found to be decreased by half in 22-day-old CRs. However, shikimate was 1.4-fold increased in 14- and 22-day-old shoots of P-deficient plants. Phenylalanine and tyrosine are precursors of the flavanone naringenin, which was increased in 22-day-old shoots (2.8-fold) and CRs (3.1-fold). Naringenin, in turn, is a precursor of plant secondary metabolites, such as flavonoids and anthocyanins.

P-deficient plants have been reported to scavenge P_i_ by degrading RNA and ATP. Such degradation might result in increased levels of nucleotides and nucleosides as degradation residues. Adenosine is a constituent of adenosine-phosphates, such as ATP, and of NADPH/NADH and RNA. Adenosine was clearly increased in all P-deficient tissues. The highest increase was found in 14-day-old shoots with a 6.1-fold increase in adenosine. Furthermore, adenine and ribose, the constituents of adenosine, exhibited elevated levels. In contrast to adenosine, the increases of adenine and ribose levels were not found in all investigated plant organs. CRs were the only plant organs with elevated levels of all three metabolites adenosine, adenine, and ribose at both harvest time points. The RNA-specific metabolite uracil did not accumulate at all in CRs. In addition, in the other plant tissues, changes in uracil levels were rather moderate, comprising an increase in 14-day-old non-CRs (1.7-fold) and 14-day-old shoots (1.3-fold) and a decrease in 22-day-old shoots (0.7-fold). None of the investigated tissues exhibited a concentration change in the uracil degradation product β-alanine.

## Discussion

### A Decline in Shoot Sugar Levels as an Early Response to P-deficiency

We have investigated the metabolic adjustments of white lupin shoots, non-CRs, and CRs to phosphorus deficiency. Metabolic profiling was used to gather new insights into the physiological adaptations of white lupin plants when phosphorus is limited. P-deficiency often results in increased shoot sugar levels (Hermans et al., 2006; Hernandez et al., 2007; Huang et al., 2008; Ganie et al., 2015). The accumulation of sugars in shoots enhances the phloem loading of sucrose and leads to sucrose translocation from shoots to roots. The delivery of sucrose from shoots is supposed to act as a P-starvation signal with the ability to induce morphological, biochemical, and gene expression changes in roots (Hammond and White, 2008, 2011). Moreover, in white lupin, sucrose is translocated from shoots to roots and acts as signaling molecule for the induction and formation of lupin CRs (Johnson et al., 1996; Liu et al., 2005; Zhou et al., 2008; Wang et al., 2015b). However, we have not found any accumulation of sugars in white lupin shoots; sugars were in contrast decreased by half compared with control plants in the 14-day-old P-deficient lupin shoots (**Figure 4**). Accumulation and translocation are processes that probably involve several hours or even days, whereas metabolic profiling provides steady-state data at a certain time point. The 14-day-old P-deficient lupins, in which shoot sugar reduction was observed had just started forming CRs. This formation is controlled by lupin shoot phosphorus status and is induced when the shoot phosphorus concentration is lower than 2–3 mg g^-1^ dry weight (Shane et al., 2003; Li et al., 2008). The accumulation of carbohydrates has been considered an early response to P-deficiency (Ciereszko et al., 2005). Phloem export of sucrose from shoots is the highest following 6 days of P-deficiency and returns to control levels after 8 days in bean plants (Cakmak et al., 1994). We have found decreased shoot sugar levels only in the younger P-deficient white lupin plants. The observed decreased sugar levels in our investigation might be the result of a preceding strong shoot-to-root translocation of sucrose that might have led to a depletion of sugar levels in shoots. In young P-deficient non-CRs and CRs, only sucrose levels were lowered, whereas fructose and glucose levels remained unaltered. CR formation, the exudation of carboxylates, and the uptake of nutrients are energy-consuming processes. The translocated sucrose might have rapidly been cleaved into fructose and glucose to cover the energy demand of the roots. Maltose, a starch degradation product, was strongly increased in young P-deficient lupin shoots. Starch biosynthesis is allosterically inhibited by Pi, which often leads to an accumulation of starch in P-deficient plants (Plaxton and Tran, 2011). An efficient usage of internal P reserves might prevent increased starch accumulation in white lupins. However, our results suggest increased starch degradation. Assuming a high energy demand of the root system, starch might be degraded to support root growth.

### Scavenging of P_i_ from Internal Sources

Under P-limiting conditions, internal organic P is a usable resource of P for plant cells. Phospholipids, small phosphorylated metabolites, and RNA are compounds containing phosphoesters from which P_i_ can be scavenged. Glucose-6-P, fructose-6-P and glycerol-3-P were strongly reduced in our investigation. These metabolites are important intermediates in glycolysis and components of phospholipids of biomembranes. Glycolysis involves phosphorylation steps but is not necessarily hindered by P-deficiency, as plants possess bypass reactions to maintain cell functions (Plaxton and Podestá, 2006; Plaxton and Tran, 2011). Plant metabolism can rely on an inorganic pyrophosphate (PP_i_)-dependent glycolysis instead of P_i_-dependent glycolysis. Inorganic pyrophosphate is formed as a byproduct of anabolic reactions (such as protein synthesis) that are essential and still take place, even under severe P-deficiency. Enzymes involved in glycolytic bypass reactions under P-deficiency, such as sucrose synthase (SuSy) and phosphoenolpyruvate carboxylase (PEPC) have been found to have higher activities and upregulated transcripts in white lupin CRs (Uhde-Stone et al., 2003; Wang et al., 2014), suggesting that glycolysis is maintained in P-deficient lupin roots. This enables lupin roots to supply the TCA cycle with carbon and to produce organic acids for exudation. Nevertheless, glucose-6-P and fructose-6-P are also generated in PP_i_-dependent glycolysis. Possibly, they are metabolized faster under P-deficiency, thus explaining the strongly decreased levels that we measured. The decreased levels of glycerol-3-P, however, are likely to be the result of a reduced biosynthesis of glycerol-3-P. Glycerol-3-P is a structural element of phospholipids, which are replaced with sulfo- and galactolipids under P-deficiency (Plaxton and Tran, 2011; Lambers et al., 2012).

Ribose, adenine, and adenosine are components of purine nucleotides and are essential elements in RNA and in ATP. These metabolites exhibited elevated levels in P-deficient lupin shoots, non-CRs, and CRs in our investigation. RNA represents the largest organic P pool in plants (Veneklaas et al., 2012). Despite the essential role of RNAs in protein biosynthesis, white lupin CRs have been found to decrease their total RNA concentration (Massonneau et al., 2001). Moreover, decreased nucleotide synthesis and increased purine nucleotides degradation has been reported for lupin CRs (Wang et al., 2014). The accumulation of ribose, adenine, and adenosine in P-deficient lupin tissues is probably the result of processes such as the degradation of RNA and purine nucleotides. Ribose, adenine, and adenosine are also components of ATP, which is known to experience sharp decreases in P-deficient plant cells (Ashihara et al., 1988; Duff et al., 1989). Depleted intracellular levels of P_i_ inhibit ADP/ATP generation, and ATP consuming glycolytic steps are bypassed by alternative adenylate-independent pathways. This might additionally contribute to the observed increased levels of free ribose, adenine, and adenosine. The RNA-specific nucleic acid uracil did not accumulate at all in CRs. In shoots and non-CRs, only moderately increased levels of uracil were observed. RNA degradation, purine nucleotide degradation, and decreased nucleotide synthesis depend on the developmental stage of CRs, as these processes are found preferentially in older CRs. Thus, uracil might have been scavenged from older tissues and been transported to younger, growing tissues.

Lupin CRs produce large amounts of organic acids, mainly citrate, which are released into the rhizosphere. The amount of carbon exuded as organic acids can range from 10% to more than 25% of plant dry weight (Vance et al., 2003). The TCA cycle in lupin CRs proceeds very differently from the TCA cycle in P-deficient non-CRs. The biosynthesis of citrate is increased in CRs, whereas the turnover of citrate and *cis*-aconitate to isocitrate is inhibited. An accumulation of citrate in CRs is the result of both increased synthesis and inhibited turnover occurring together (Neumann et al., 1999; Neumann and Martinoia, 2002; Kihara et al., 2003). Our results are consistent with these findings, as we have found a strong accumulation of citrate and *cis*-aconitate in CRs and a decrease in isocitrate. Neither shoots nor non-CRs showed similarly changed levels in those metabolites. The accumulation of succinate and fumarate in CRs, non-CRs, and shoots might represent a slowed turnover of those organic acids.

### Accumulation of Amino Acids and Stimulation of Secondary Metabolism

About half of the measured amino acids accumulated under P-deficiency. An increase in free amino acids might be the result of protein degradation and a suppressed protein biosynthesis, as is assumed by other investigators (Huang et al., 2008; Pant et al., 2014). A strong increase in certain amino acids, such as asparagine, has been previously described, and an altered biosynthesis or incorporation into proteins has been suggested as being responsible for the strong reaction of specific amino acids (Johnson et al., 1996). The rare aromatic amino acid tryptophan showed the strongest accumulation of all amino acids in our investigation. CRs of 22-day-old lupin plants had 19-fold higher tryptophan levels than the roots of P-fed plants. An almost identical accumulation of tryptophan in roots has been reported for P-deficient *A. thaliana* (Pant et al., 2014). During those experiments, *A. thaliana* plants grown for 16 days under low phosphorus conditions had 20-times higher tryptophan levels in their roots than found in the control plants. There might be a connection between the accumulation of free tryptophan in P-deficient roots and the morphological adaptations of roots to P-deficiency. Plants use tryptophan in the synthesis of auxin (Mano and Nemoto, 2012), and root-borne auxins are supposed to be involved in CR formation (Meng et al., 2013; Wang et al., 2015a). The aromatic amino acids phenylalanine and tyrosine and the flavanone naringenin are part of the phenylpropanoid pathway by which flavonoids are synthesized. All of these metabolites were increased by approximately threefold in 22-day-old shoots or CRs. White lupin roots release flavonoids and isoflavonoids into the rhizosphere (Weisskopf et al., 2006b). Flavonoids not only prevent microbial degradation of the exuded organic acids (Weisskopf et al., 2006a), but are also directly involved in the mobilization of soil phosphorus (Tomasi et al., 2008). Naringenin is involved in the biosynthesis of anthocyanins, which are known to accumulate in plant shoots under P-deprivation (Ticconi et al., 2001; Varadarajan et al., 2002) and are supposed to have a photoprotective function (Steyn et al., 2002; Zeng et al., 2010).

## Conclusion

**FIGURE 5:**
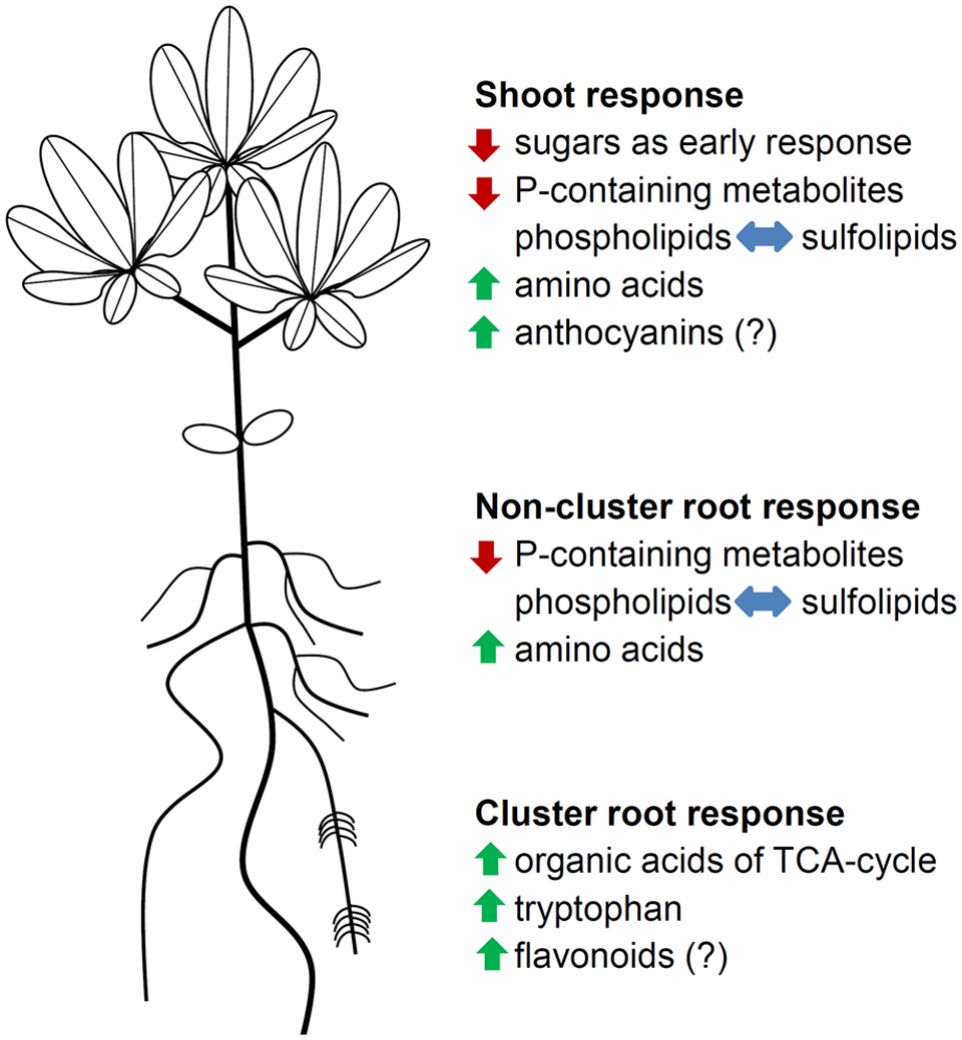
**Schematic summary of metabolic and physiological adjustments in P-deficient white lupins**. Green arrows indicate an increase in metabolite levels, red arrows indicate a decrease in metabolite levels, and blue arrows indicate a replacement of phospholipids with sulfolipids. Metabolic groups with (?) were not determined.

Phosphorus deficiency exerts a great impact on white lupin metabolism, leading to a wide range of metabolic and physiological adjustments (**Figure 5**). Young P-deficient lupin plants exhibit a decline in sugar concentrations in the shoot, suggesting an adaption of carbohydrate partitioning between shoot and root as an early response to the limited available P. P-deficient lupins scavenge internal P_i_ from small P-containing metabolites and from phospholipids. Protein anabolism is reduced under P-deficiency, but the formation of secondary metabolites is enhanced. The biosynthesis of organic acids is increased in CRs, with citrate showing the largest accumulation.

## Conflict of Interest Statement

The authors declare that the research was conducted in the absence of any commercial or financial relationships that could be construed as a potential conflict of interest.
